# Supplementary material for: The USP46 complex deubiquitylates LRP6 to promote Wnt/β-catenin signaling
Source: Nat Commun. 2023 Oct 5;14:6173. doi: 10.1038/s41467-023-41836-z (PMC10556042; doi:10.1038/s41467-023-41836-z)
Supplement: Supplementary file 1 — Supplementary Information [file 41467_2023_41836_MOESM1_ESM.pdf]

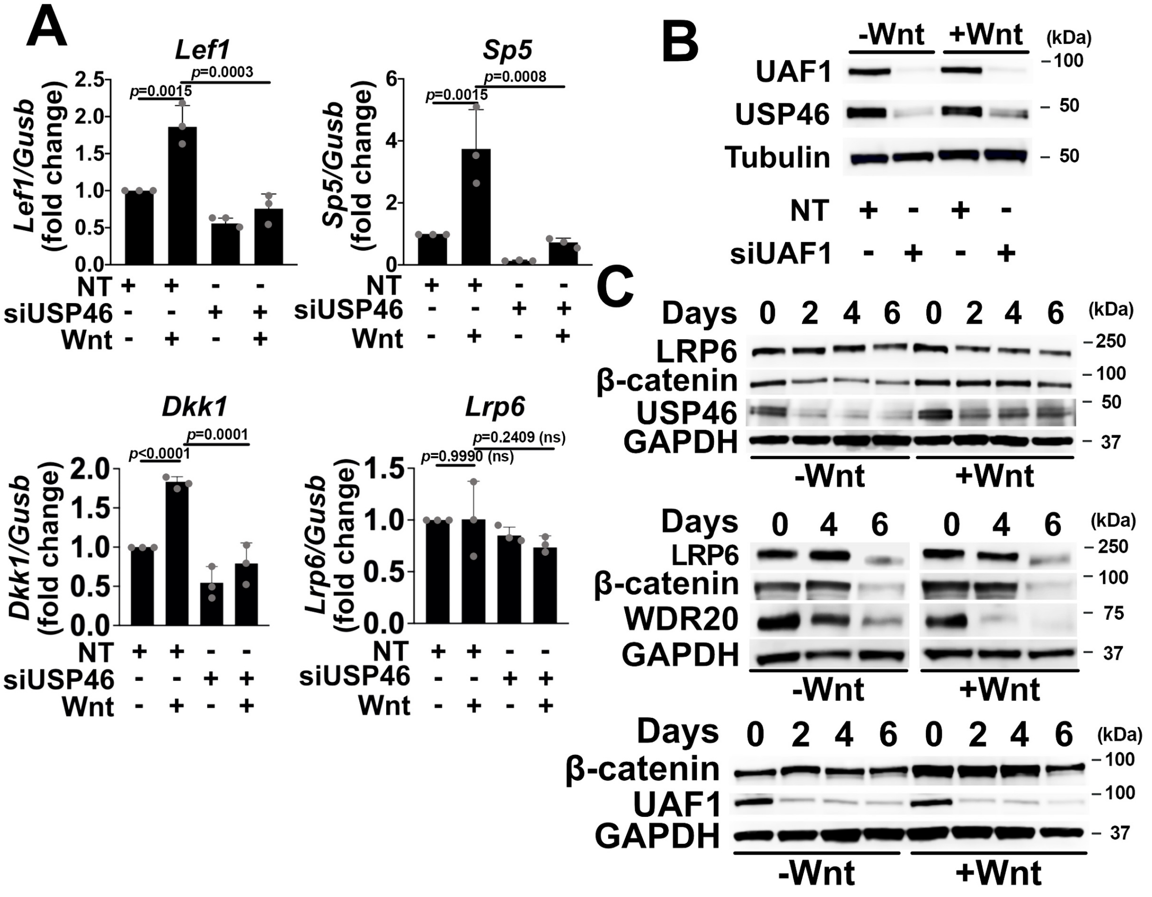

Supplementary Figure 1. USP46 promotes Wnt signaling upstream of the β-catenin degradation complex. (**A**) Quantitative real-time RT-PCR of endogenous Wnt target genes and *Lrp6* in HEK293 treated with USP46 siRNAs or non-targeting (NT) control. Graphs show a ratio relative to *Gusb* (control). Results (mean ±SD) of three independent real-time RT-PCR reactions are shown. p-values compared Wnt3a-treated NT to non-Wnt treated NT and Wnt3a-treated USP46 siRNA. $p \geq 0.05$ is not significant (ns). Significance was analyzed by one-way ANOVA followed up with Tukey's multiple comparisons test. (**B**) Knockdown of UAF1 decreased levels of USP46. HEK293 cells were transfected with non-targeting (NT) or pooled UAF1 siRNAs, and immunoblotting for USP46 and UAF1 was performed. (**C**) Knockout of USP46, WDR20, and UAF1 by CRISPR-Cas9 editing decreased β-catenin and LRP6 levels. HEK293T cells stably expressing Cas9 were transfected with tracrRNA and crRNAs targeting individual components of the USP46 complex. Cells were treated with or without Wnt3a (10ng/ml), and lysates were collected for immunoblotting on the indicated days. Graphs and immunoblots are representative of at least three independent experiments. Source data are provided as a Source Data file.

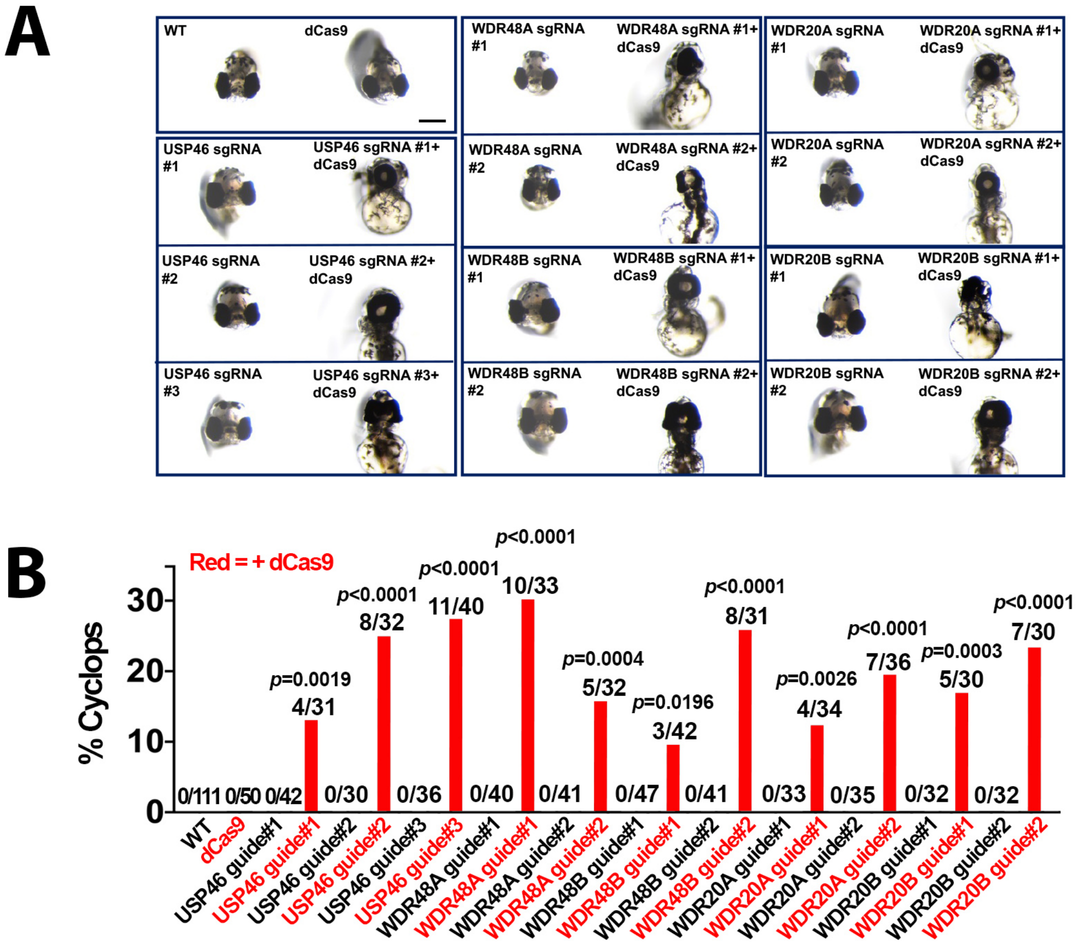

Supplementary Figure 2. CRISPRi of USP46 complex in zebrafish induces a cyclopic phenotype.

(**A**) Representative images are shown on top. Scale bar = 200 mm. (**B**) The percentage of cyclopic embryos is graphed with absolute numbers indicated on the top of each bar. Graphs show mean ± SEM. p-values compare guide RNA plus dCas9-injected embryos versus WT. Significance was assessed using 2-sided Fisher's exact test. n = 3 independent pools of embryos. Comparisons between Wildtype (WT) versus dCas9, WT versus guide RNA alone, and dCas9 versus guide RNA alone were not statistically significant. Source data are provided as a Source Data file.

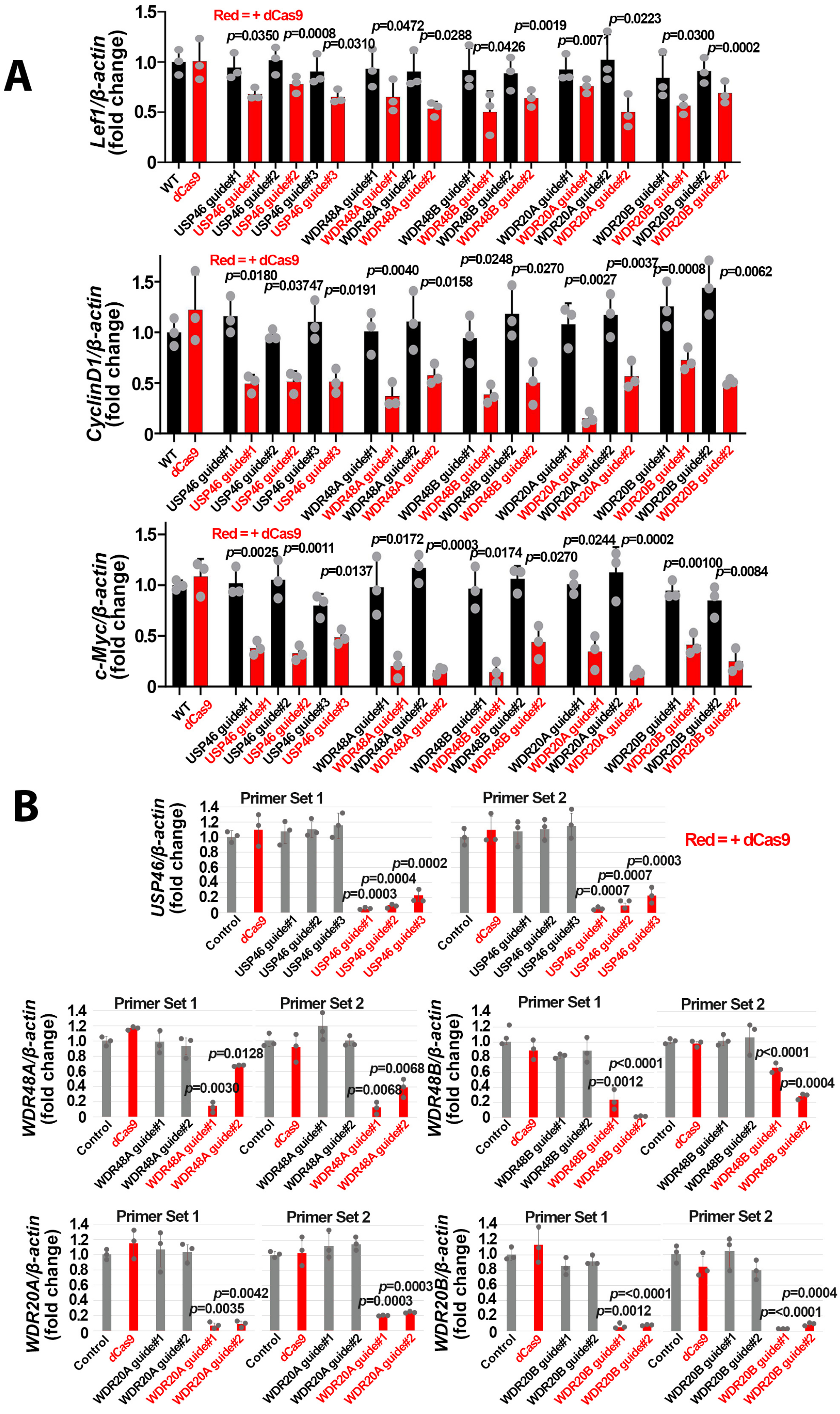

A
Red = + dCas9
Lef1/β-actin (fold change)
p=0.0350
p=0.0008
p=0.0310
p=0.0472
p=0.0288
p=0.0426
p=0.0019
p=0.0071
p=0.0223
p=0.0300
p=0.0002
CyclinD1/β-actin (fold change)
p=0.0180
p=0.03747
p=0.0191
p=0.0040
p=0.0158
p=0.0248
p=0.0270
p=0.0027
p=0.0037
p=0.0008
p=0.0062
c-Myc/β-actin (fold change)
p=0.0025
p=0.0011
p=0.0137
p=0.0172
p=0.0003
p=0.0174
p=0.0270
p=0.0244
p=0.0002
p=0.00100
p=0.0084
WT
dCas9
USP46 guide#1
USP46 guide#2
USP46 guide#3
WDR48A guide#1
WDR48A guide#2
WDR48B guide#1
WDR48B guide#2
WDR20A guide#1
WDR20A guide#2
WDR20B guide#1
WDR20B guide#2
B
Red = + dCas9
Primer Set 1
Primer Set 2
USP46/β-actin (fold change)
p=0.0003
p=0.0004
p=0.0002
p=0.0007
p=0.0007
p=0.0003
WDR48A/β-actin (fold change)
p=0.0030
p=0.0128
p=0.0068
p=0.0068
WDR48B/β-actin (fold change)
p=0.0012
p<0.0001
p<0.0001
p=0.0004
WDR20A/β-actin (fold change)
p=0.0035
p=0.0042
p=0.0003
p=0.0003
WDR20B/β-actin (fold change)
p=0.0012
p=<0.0001
p=<0.0001
p=0.0004
Control

Supplementary Figure 3. CRISPRi of USP46 complex in zebrafish result in a reduction of Wnt target gene transcripts.

(**A**) mRNAs were isolated from injected single embryos, and *Lef1*, *CyclinD1,* and *c-Myc* levels were quantified by RT-PCR. (**B**) qRT-PCR confirms significant reductions in mRNA levels of USP46 components in their respective guide RNA plus dCas9-injected embryos. Two primer sets were used for each gene. Gene expression is graphed as a ratio to *β-actin* control. p-values compare guide RNA-injected versus guide RNA plus dCas9-injected embryos. Graphs show mean ± SEM. Significance was analyzed by two-tailed Student's *t*-test. Comparisons between Wildtype (WT) control versus dCas9, WT versus guide RNA alone, and dCas9 versus guide RNA alone were not statistically significant. Source data are provided as a Source Data file.

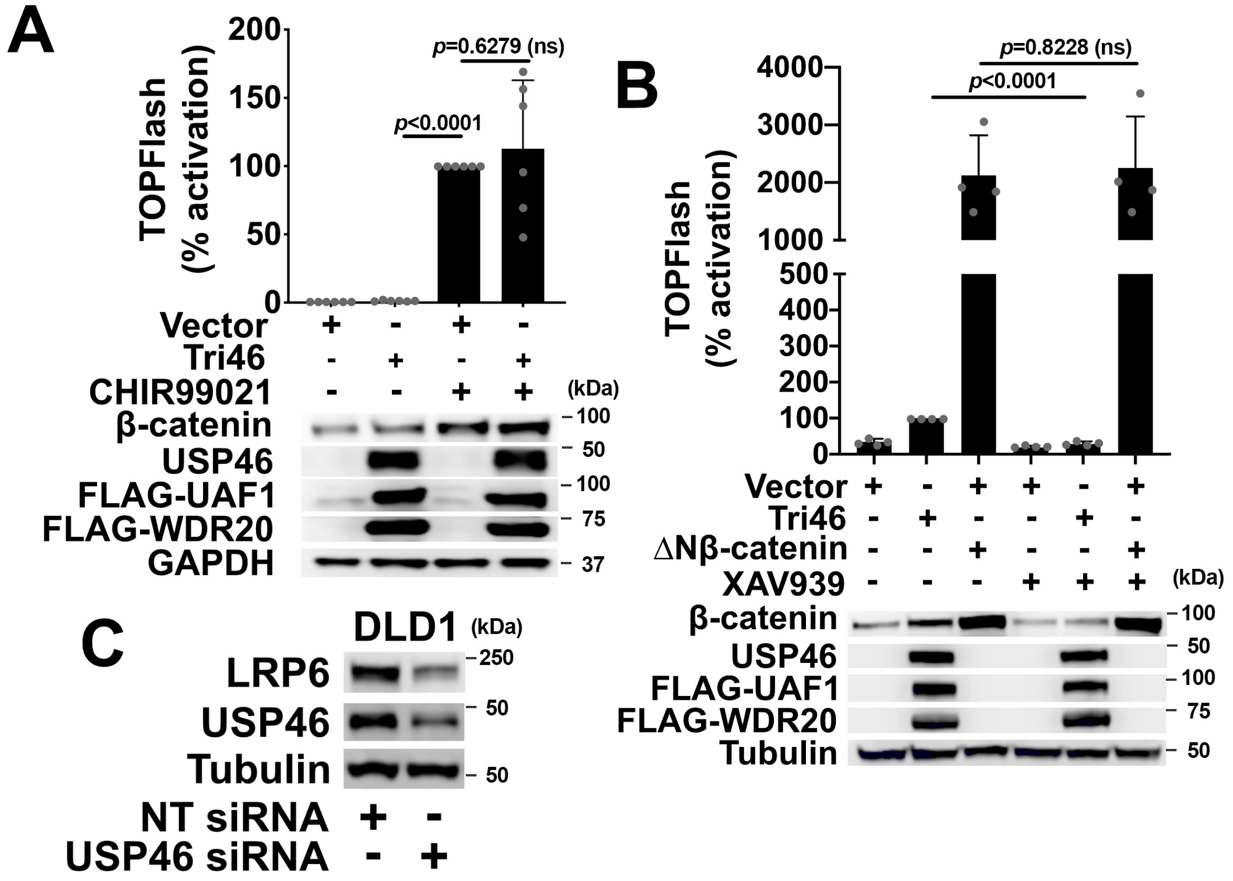

Supplementary Figure 4. The USP46 Complex act upstream of the β-catenin destruction complex.

(**A**) Activation of Wnt signaling by GSK3 inhibition is not potentiated by the USP46 complex. HEK293 STF cells were transfected with the USP46 complex and treated with CHIR99021 (2 mM). Graphs show mean ±SD of TOPFlash normalized to CHIR99021 control. p-value compares CHIR99021-treated cells with USP46 complex-transfected cells. ns compares CHIR99021-treated cells transfected with vector control or the USP46 complex. (**B**) The AXIN stabilizer, XAV939, inhibits Wnt signaling stimulated by the USP46 complex but not by a non-degradable form of β-catenin (DN-β-catenin). Cells were transfected as indicated and treated with or without XAV939 (1 mM) overnight. Graphs show mean ±SD of TOPFlash normalized to USP46 complex-transfected control. p-value compares USP46 complex-transfected cells minus or plus treatment with XAV939. ns compare DN-β-catenin-transfected cells minus or plus treatment with XAV939. Significance was analyzed by one-way ANOVA followed by Tukey's multiple comparisons test. Graphs show representatives of n = 3 independent experiments performed in triplicates. $p \geq 0.05$ is not significant (ns). (**C**) Knockdown of USP46 in the colorectal cancer cell line, DLD1, results in decreased LRP6 levels. DLD1 cells were transfected with non-targeting (NT) or pooled USP46 siRNAs. Immunoblots are representative of at least three independent experiments. Source data are provided as a Source Data file.

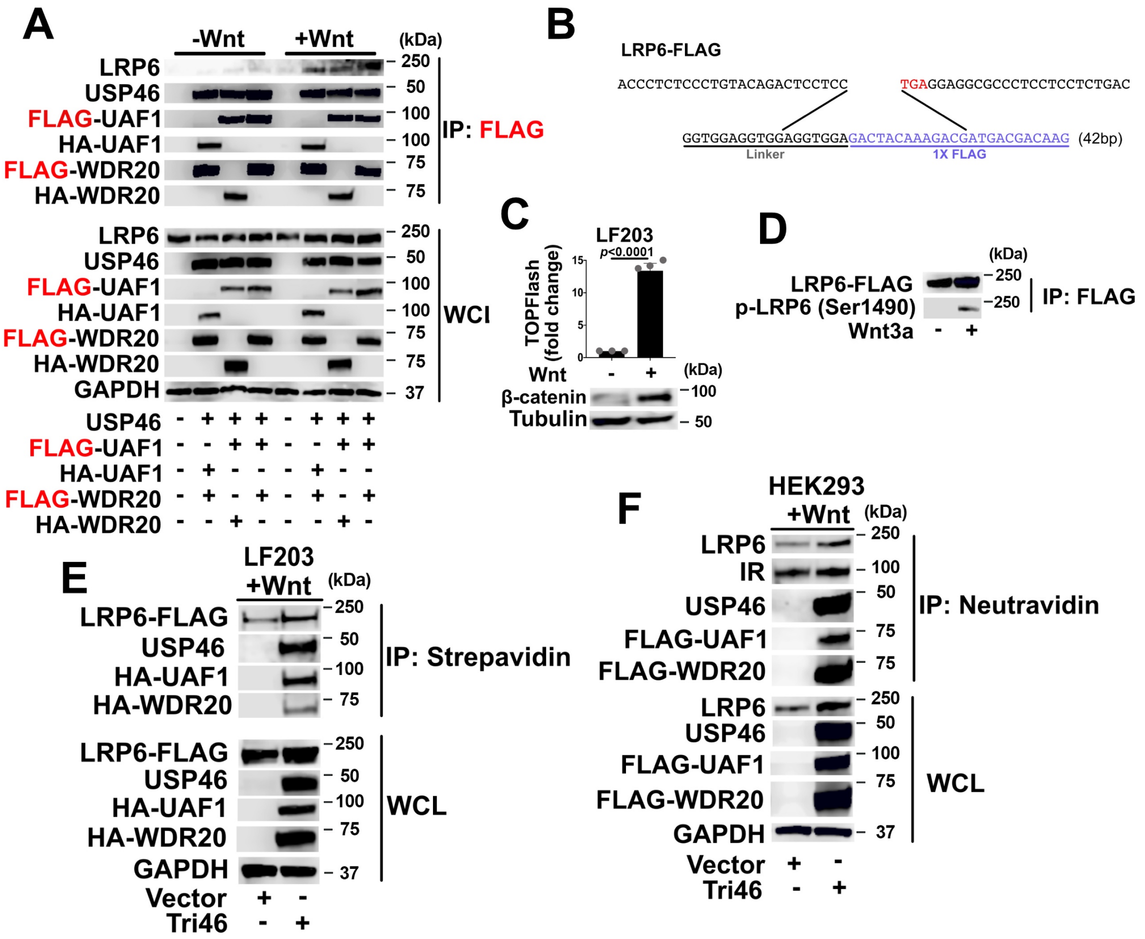

Supplementary Figure 5. The USP46 complex co-precipitates with cell surface LRP6.

(**A**) Endogenous LRP6 co-immunoprecipitates with FLAG-tagged WDR20 or UAF1. HEK293 cells were transfected as indicated and treated with Wnt3a (10ng/ml) overnight. Cells were lysed, proteins immunoprecipitated with anti-FLAG conjugated beads, and immunoblotting was performed. (**B-D**) Generation of the LF203 HEK293 cell line by CRISPR-Cas9 knock-in of a FLAG epitope into the endogenous LRP6 receptor shows normal Wnt activation. (**B**) Genomic sequencing confirms the correct in-frame insertion of the FLAG tag into endogenous LRP6. (**C**) Activation of Wnt signaling in LF203 cells by Wnt3a. Cells were transfected with plasmids encoding TOPFlash and Renilla Luciferase control reporters and treated with Wnt3a (10 ng/ml) overnight. Firefly and Renilla luciferase activities were assessed by the Dual-Glo Luciferase Assay. Graph shows mean ±SD of TOPFlash normalized to no Wnt3a control. p-value compares non-treated and Wnt3a-treated cells. Significance was analyzed by two-tailed Student's *t*-test. (**D**) LRP6-FLAG is activated by Wnt3a in LF203 cells. LF203 cells were treated overnight with or without Wnt3a, lysed, and subjected to immunoprecipitation with anti-FLAG conjugated beads. Samples were analyzed by immunoblotting for total LRP6 and p-LRP6 (Ser1490), which detects the activated receptor. (**E-F**) The USP46 complex is pulled down in a cell surface biotinylation assay. Cells transfected with the USP46 complex and treated overnight with Wnt3a were subjected to surface biotinylation, lysis, avidin-pulldown, and immunoblotting. (**E**) The USP46 complex co-precipitates with LRP6-FLAG in LF203 cells and (**F**) LRP6 in HEK293 cells. GAPDH and Tubulin are loading control. Graph and immunoblots are representative of at least three independent experiments. Source data are provided as a Source Data file.

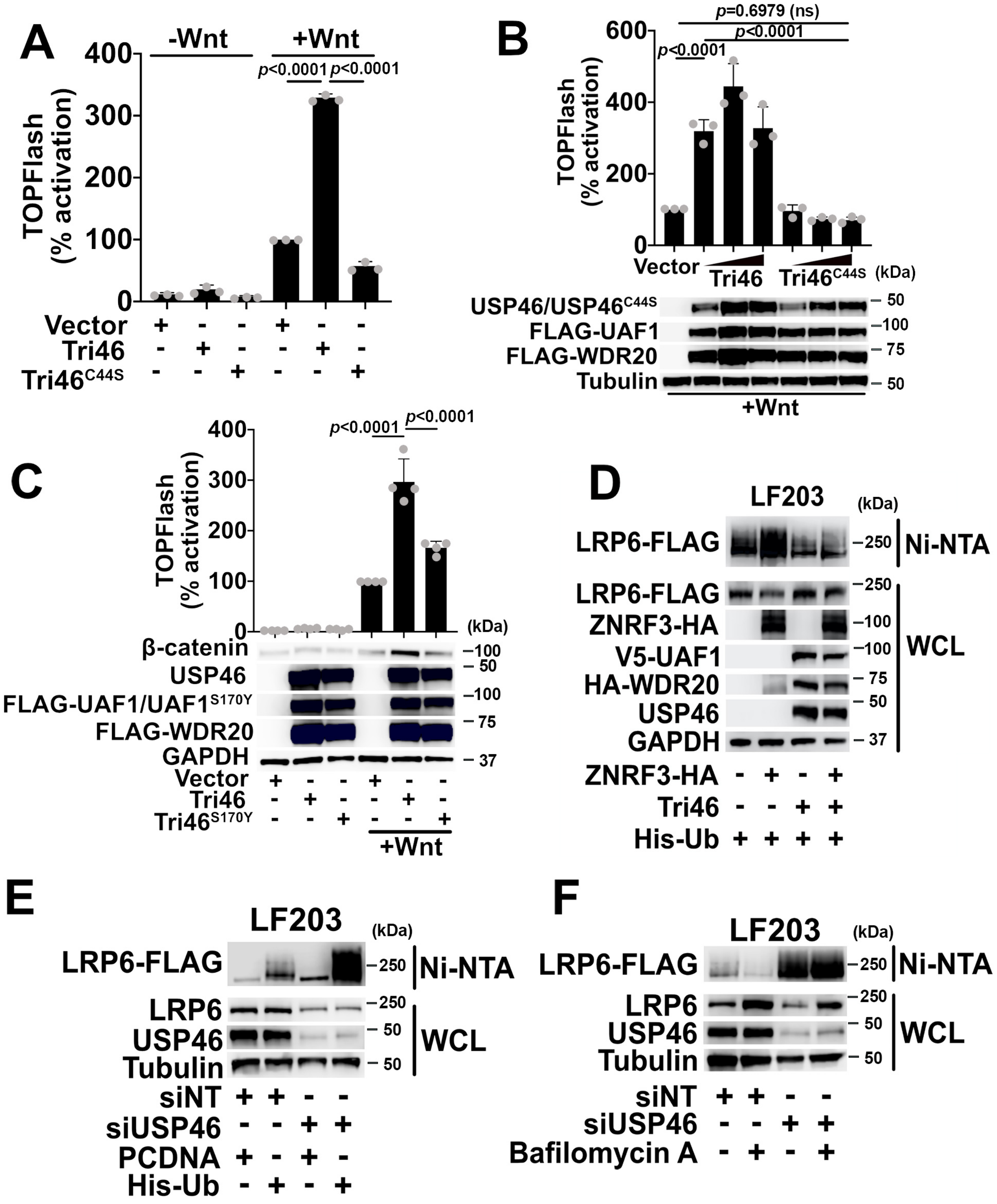
A
-Wnt
+Wnt
p<0.0001
p<0.0001
TOPFlash (% activation)
Vector
Tri46
Tri46C44S
B
p=0.6979 (ns)
p<0.0001
p<0.0001
TOPFlash (% activation)
Vector
Tri46
Tri46C44S
(kDa)
USP46/USP46C44S
FLAG-UAF1
FLAG-WDR20
Tubulin
+Wnt
C
p<0.0001
p<0.0001
TOPFlash (% activation)
(kDa)
β-catenin
USP46
FLAG-UAF1/UAF1S170Y
FLAG-WDR20
GAPDH
Vector
Tri46
Tri46S170Y
+Wnt
D
LF203
(kDa)
LRP6-FLAG
Ni-NTA
LRP6-FLAG
ZNRF3-HA
V5-UAF1
HA-WDR20
USP46
GAPDH
WCL
ZNRF3-HA
Tri46
His-Ub
E
LF203
(kDa)
LRP6-FLAG
Ni-NTA
LRP6
USP46
Tubulin
WCL
siNT
siUSP46
PCDNA
His-Ub
F
LF203
(kDa)
LRP6-FLAG
Ni-NTA
LRP6
USP46
Tubulin
WCL
siNT
siUSP46
Bafilomycin A

Supplementary Figure 6. The deubiquitylase activity of the USP46 complex is required for its regulation of the Wnt pathway.

(**A-C**) HEK293 STF cells were transfected and treated with recombinant Wnt3a (10 ng/ml) for 24 hr as indicated. Lysates were then prepared for TOPFlash and immunoblotting. (**A**) Overexpression of catalytically dead USP46^{C44S} fails to potentiate Wnt signaling as assessed by Wnt reporter assay. p-values compare USP46 complex (Tri46) and USP46^{C44S} complex (Tri46^{C44S}) transfected cells in the presence of Wnt3a. (**B**) Titration of increasing amounts of catalytically dead USP46^{C44S} fails to potentiate Wnt signaling as assessed by Wnt reporter assay. p-values compare 1) cells transfected with vector with cells transfected with the lowest amount of USP46 and 2) cells transfected with the lowest amount of USP46 and cells transfected with the highest amount of USP46^{C44S}. ns compares vector-transfected cells with the highest amount of USP46^{C44S} complex-transfected cells. $p \geq 0.05$ is not significant (ns). (**C**) The USP46/UAF1^{S170Y}/WDR20 (Tri46^{S170Y}) complex exhibits reduced activity. p-values compare 1) Wnt3a-treated cells transfected with wild-type UAF1 USP46 complex (Tri46) or catalytically dead UAF1^{S170Y} USP46 complex (Tri46^{S170Y}) and 2) Wnt3a-treated cells transfected with vector or Tri46 complex. All graphs show mean $\pm$SD of TOPFlash normalized to vector transfected, Wnt3a-treated control. Significance was analyzed by one-way ANOVA followed by Tukey's multiple comparisons test. (**D-F**) His-ubiquitylation assays. LF203 cells were transfected as indicated, lysed under denaturing conditions, and His-Ub modified proteins isolated by nickel affinity purification. LRP6 and FLAG-tagged proteins were detected by immunoblotting with anti-LRP6 and anti-Flag antibodies, respectively. WCL = whole cell lysates. (**D**) Overexpression of ZNRF3 in LF203 cells promotes LRP6 ubiquitylation, which is opposed by overexpression of the USP46 complex. (**E**) Detection of ubiquitylated LRP6 with the His-ubiquitylation assay is specific to cells transfected with His-Ub. PCNDA is transfection control. (**F**) Treatment with Bafilomycin A blocks LRP6 turnover upon USP46 knockdown. Cells were transfected as indicated and treated with DMSO or Bafilomycin A (10 nM) overnight prior to the His-ubiquitylation assay. Graphs show representatives of n = 3 independent experiments performed in triplicates. Immunoblots are representative of at least three independent experiments. Source data are provided as a Source Data file.

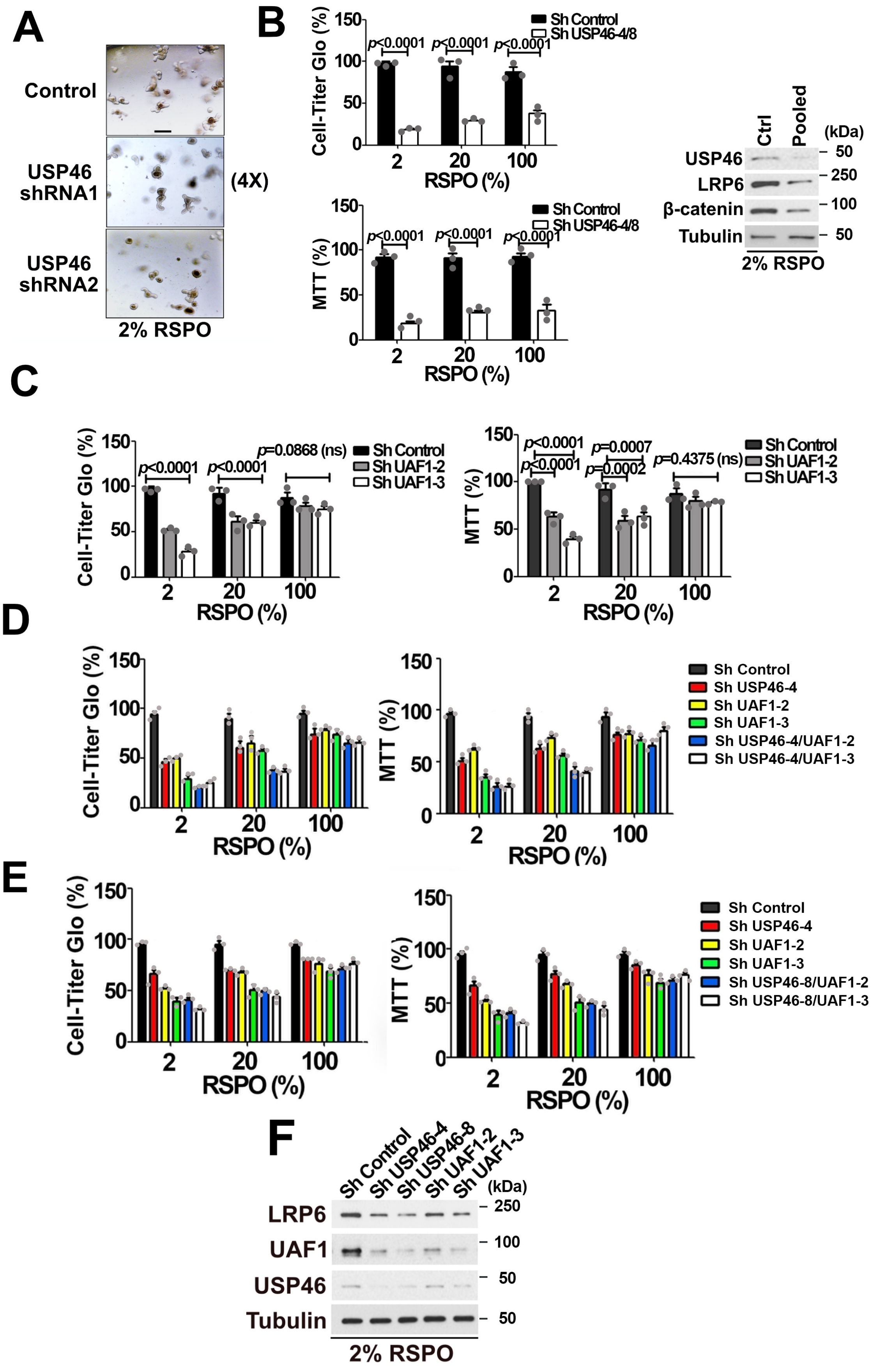
A
Control
USP46 shRNA1
(4X)
USP46 shRNA2
2% RSPO
B
Cell-Titer Glo (%)
Sh Control
Sh USP46-4/8
p<0.0001
RSPO (%)
MTT (%)
Ctrl
Pooled
(kDa)
USP46
LRP6
β-catenin
Tubulin
2% RSPO
C
p=0.0868 (ns)
Sh UAF1-2
Sh UAF1-3
p=0.0007
p=0.0002
p=0.4375 (ns)
D
Sh USP46-4
Sh USP46-4/UAF1-2
Sh USP46-4/UAF1-3
E
Sh USP46-8/UAF1-2
Sh USP46-8/UAF1-3
F
Sh USP46-8
UAF1

Supplementary Figure 7. The viability of wild-type intestinal organoids is dependent on USP46 and UAF1. (**A-F**) Knockdown of USP46 and/or UAF1 decreases wild-type intestinal organoid viability. Organoids were infected with control lentivirus or independent lentiviruses targeting USP46 and/or UAF1. (**A**) Representative images of intestinal organoids treated with USP46 shRNA 1 or 2 and grown in the presence of 2% RSPO-CM. Scale bar = 200 mM. (**B**) Intestinal organoids infected with a control lentivirus or pooled lentiviruses expressing USP46 shRNAs were grown with an increasing RSPO-conditioned media (CM). Viability was assessed by both Cell-Titer Glo and MTT assays. Graph shows mean $\pm$SD of Cell-Titer Glo and MTT assay, respectively. p-values compare control-treated with pooled USP46 shRNAs. (Right) Immunoblot shows decreased USP46, LRP6, and β-catenin in wildtype organoids with pooled USP46 shRNA treatment. (**C**) The sensitivity of intestinal organoids to UAF1 depletion is RSPO-dependent. Intestinal organoids infected with a control lentivirus or two independent lentiviruses expressing UAF1 shRNAs were grown with a decreasing RSPO-conditioned media (CM). Viability was assessed by Cell-Titer Glo (top) and MTT assay (bottom). Graph shows mean $\pm$SD of Cell-Titer Glo and MTT assay, respectively. p-values compare control treatment with UAF1 shRNA 1 or 2 treatment. (**D-F**) Intestinal organoids infected with a control lentivirus or independent lentiviruses targeting USP46 and/or UAF1 were grown with a decreasing RSPO CM. (**D-E**) Depletion of USP46 and UAF1 results in a greater decrease in cell viability upon reduction of RSPO CM. Viability was assessed by Cell-Titer Glo (left) and MTT assay (right). Graph shows mean $\pm$SD of Cell-Titer Glo and MTT assay, respectively. (**D**) p-values compare to Sh Controls using Cell-Titer Glo are as follows: <0.0001 for all conditions at 2% RSPO-CM; 0.0002 (Sh USP46-4), 0.0018 (Sh UAF1-2), <0.0001 (Sh UAF1-3), <0.0001 (sh USP46-4/UAF1-2), and <0.0001 (sh USP46-4/UAF1-3) at 20% RSPO-CM; 0.0139 (Sh USP46-4), 0.0756 (ns) (Sh UAF1-2), 0.0120 (Sh UAF1-3), 0.0001 (sh USP46-4/UAF1-2), and 0.0002 (sh USP46-4/UAF1-3) at 100% RSPO-CM. p-values compare to Sh Controls using MMT are as follows: <0.0001 for all conditions at 2% RSPO-CM; <0.0001 (Sh USP46-4), 0.0029 (Sh UAF1-2), <0.0001 (Sh UAF1-3), <0.0001 (sh USP46-4/UAF1-2), and <0.0001 (sh USP46-4/UAF1-3) at 20% RSPO-CM; 0.0128 (Sh USP46-4), 0.0183 (Sh UAF1-2), 0.0009 (Sh UAF1-3), <0.0001 (sh USP46-4/UAF1-2), and 0.0801 (ns) (sh USP46-4/UAF1-3) at 100% RSPO-CM.

(**E**) p-values compare to Sh Controls using Cell-Titer Glo are as follows: <0.0001 for all conditions at 2% RSPO-CM; <0.0001 for all conditions at 20% RSPO-CM; 0.0056 (Sh USP46-8), 0.0002 (Sh UAF1-2), <0.0001 (Sh UAF1-3), <0.0001 (sh USP46-8/UAF1-2), and 0.0002 (sh USP46-8/UAF1-3) at 100% RSPO-CM. p-values compare to Sh Controls using MMT are as follows: <0.0001 for all conditions at 2% RSPO-CM; 0.0020 (Sh USP46-8), <0.0001 (Sh UAF1-2), <0.0001 (Sh UAF1-3), <0.0001 (sh USP46-8/UAF1-2), and <0.0001 (sh USP46-8/UAF1-3) at 20% RSPO-CM; 0.2338 (ns) (Sh USP46-8), 0.0013 (Sh UAF1-2), <0.0001 (Sh UAF1-3), <0.0001 (sh USP46-8/UAF1-2), and 0.0013 (sh USP46-8/UAF1-3) at 100% RSPO-CM. p-value $\geq$ 0.05 is not significant (ns). Significance for all graphs were analyzed by one-way ANOVA followed by Tukey's multiple comparisons test. All graphs are representative of n = 3 independent experiments performed in triplicates. (**F**) Immunoblot confirms depletion of USP46 and UAF1 in organoids treated with shRNA lentiviruses. Furthermore, knockdown of USP46 and UAF1 destabilizes UAF1 and USP46, respectively. Immunoblots are representative of three independent experiments. Source data are provided as a Source Data file.

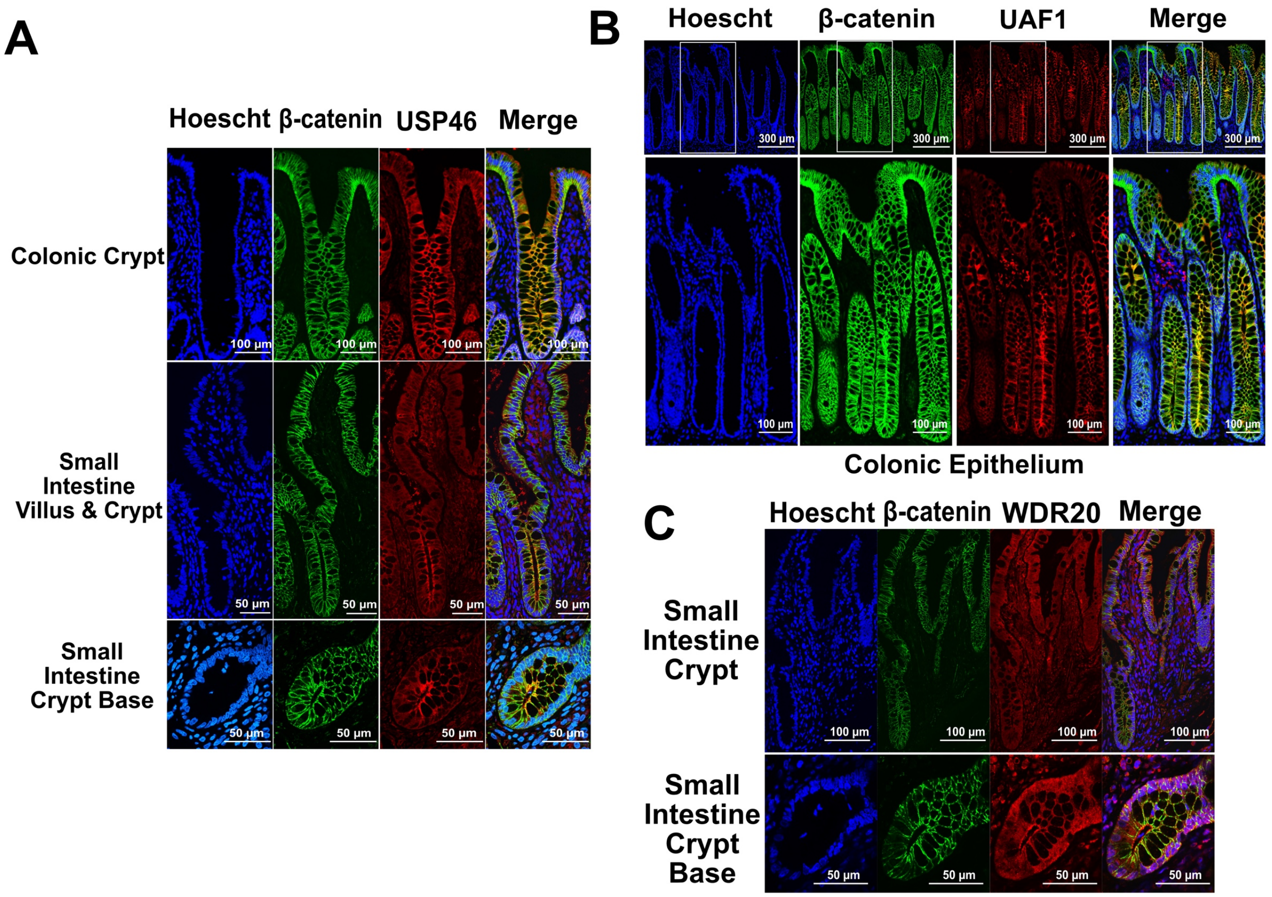

Supplementary Figure 8. Membranous USP46 and UAF1 are concentrated in the crypts, while WDR20 is uniform in the intestine.

(**A-C**) Multiplex immunofluorescence of USP46, UAF1, and WDR20 FFPE human tissue blocks of human small intestine and colon. (**A**) USP46 immunostaining increases in intensity toward the base of the intestinal crypts. (**B**) UAF1 is present in the epithelium of intestinal crypts. (**C**) WDR20 is uniformly expressed in the human small intestine and colon. Hoescht stains the nucleus. n=3 independent experiments. Source data are provided as a Source Data file.

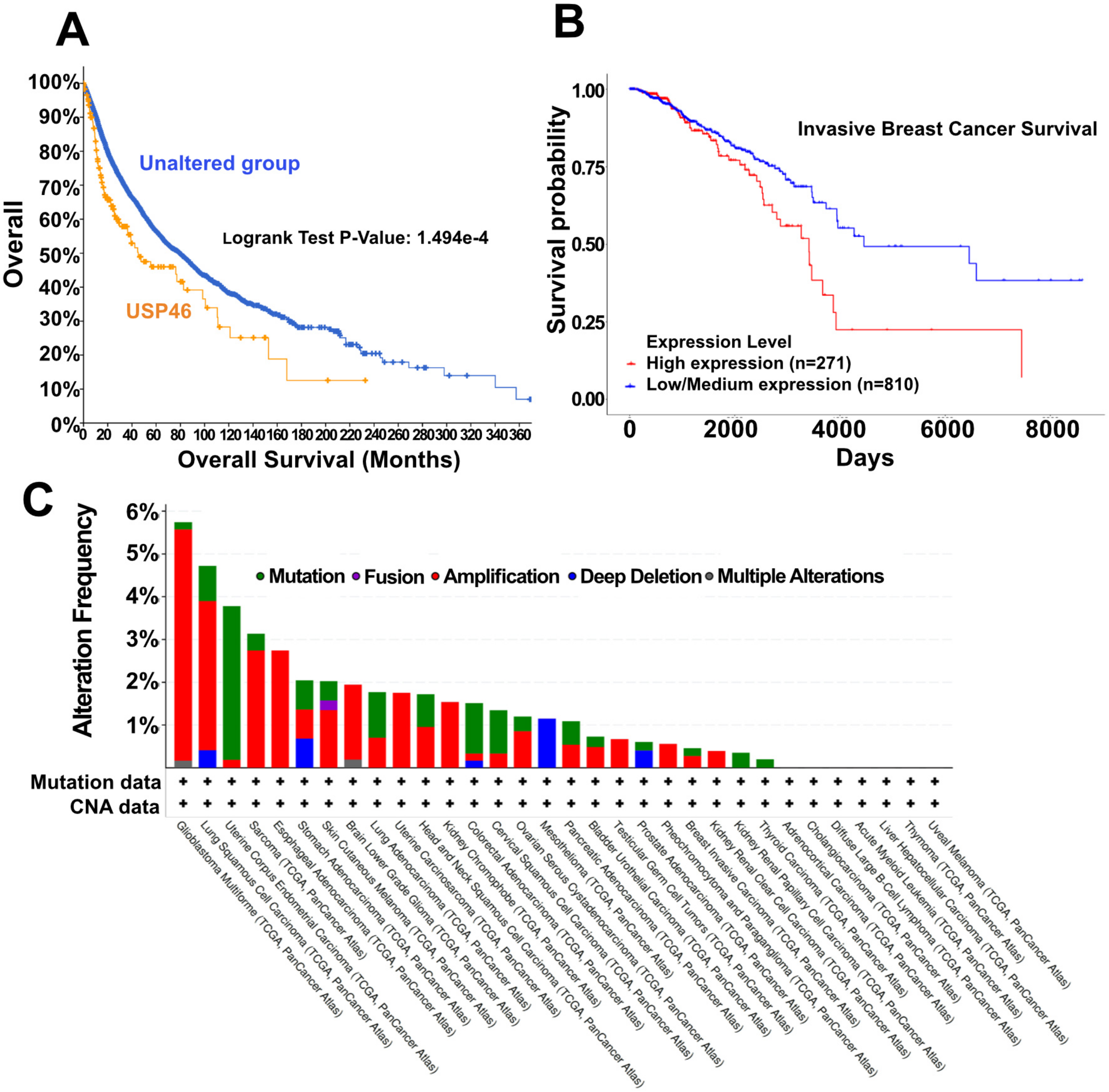

Supplementary Figure 9. USP46 expression is upregulated in cancer and associated with a poorer prognosis. (**A**) Altered USP46 expression in cancer is associated with decreased overall survival. The median overall survival of patients with USP46 alterations was 45.14 months (N=178) compared to 79.46 months (N=10,623) for the unaltered group across tumor types. (**B**) High USP46 mRNA expression levels in the TCGA correlate with decreased overall survival in invasive breast cancer (p-value $\leq$ 0.02). Decreased survival was also observed for liver hepatocellular carcinoma (p-value $\leq$ 0.02) and sarcoma (p-value $\leq$ 0.05). (**C**) Gene amplification of USP46 is commonly observed in human cancers. Altered group: fusions, deletions, amplifications, and mutations. CNA, copy number alteration.

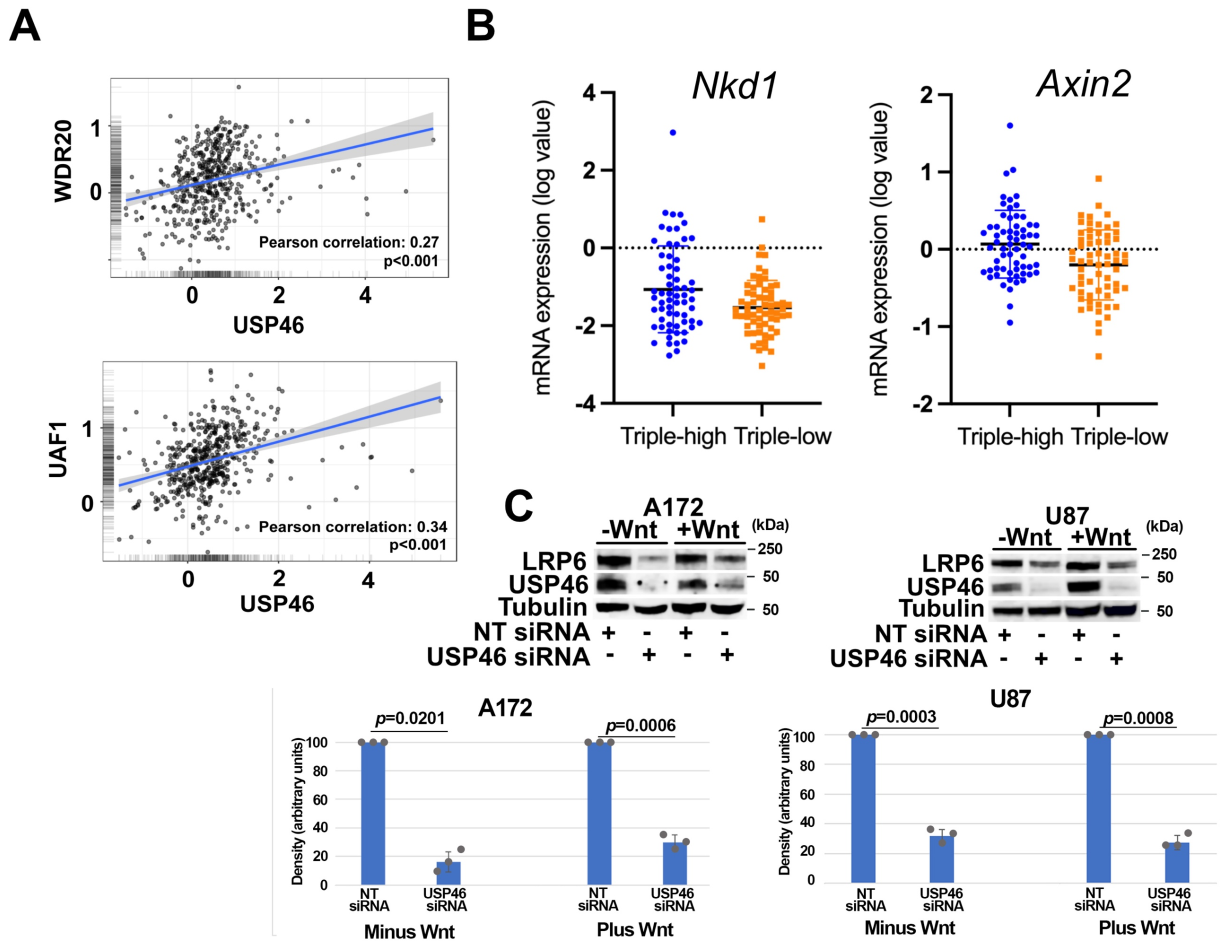

Supplementary Figure 10. Elevated USP46 complex levels are associated with increased Wnt activity in glioblastoma.

(A) USP46 positively correlates with the expression of UAF1 and WDR20 in glioblastoma (TCGA-GBM Agilent platform). Correlation analysis between indicated genes (Top, USP46, and WDR20 and Bottom, USP46, and UAF1) was performed using the TCGA-GBM mRNA expression data from the Agilent-4502A platform (http://gliovis.bioinfo.cnio.es/). Pearson correlation coefficient and p-value were calculated and indicated in each panel. (**B**) mRNA expression levels of Wnt target genes, *Nkd1* (p-value = 0.0057) and *Axin2* (p-value = 0.0010), in the USP46-high/UAF1-high/WDR20-high group (triple-high) and the USP46-low/UAF1-low/WDR20-low group (triple-low) groups. (**C**) USP46 is required for the stabilization of LRP6 in glioblastoma cell lines. siRNA knockdown of USP46 decreased steady-state levels of LRP6 in A172 and U87 glioblastoma cell lines. LRP6 levels were quantified and normalized to the NT control. Graph shows mean ± SEM of three independent replicates. p-values (two-tailed Student's *t*-test) versus NT control. Immunoblots are representative of three independent experiments. Source data are provided as a Source Data file.

sgRNA	Sequence
USP46 guide #1	UUCAUAAAGAUGGCAACGAGGUUUUAGAGCUAGAAAUAGCAAGUUAAAAUAAGGCUAGUCCGUUAUCAACUUGAAAAAGUGGCACCGAGUCGGUGCUUUU
USP46 guide #2	UCUACUUACCAUAUUACAAAGUUUUAGAGCUAGAAAUAGCAAGUUAAAAUAAGGCUAGUCCGUUAUCAACUUGAAAAAGUGGCACCGAGUCGGUGCUUUU
USP46 guide #3	ACGUAAUGAUGAUGGUUGAAGUUUUAGAGCUAGAAAUAGCAAGUUAAAAUAAGGCUAGUCCGUUAUCAACUUGAAAAAGUGGCACCGAGUCGGUGCUUUU
WDR48A guide #1	CACGGCGUCAACAUGUAAGAGUUUUAGAGCUAGAAAUAGCAAGUUAAAAUAAGGCUAGUCCGUUAUCAACUUGAAAAAGUGGCACCGAGUCGGUGCUUUU
WDR48A guide #2	UCUUCUUGGCGUGACGGAAGGUUUUAGAGCUAGAAAUAGCAAGUUAAAAUAAGGCUAGUCCGUUAUCAACUUGAAAAAGUGGCACCGAGUCGGUGCUUUU
WDR48B guide #1	AUUAUCUGUAAAUUAUCACCGUUUUAGAGCUAGAAAUAGCAAGUUAAAAUAAGGCUAGUCCGUUAUCAACUUGAAAAAGUGGCACCGAGUCGGUGCUUUU
WDR48B guide #2	CUGAUUGUCAACAUGCAAGAGUUUUAGAGCUAGAAAUAGCAAGUUAAAAUAAGGCUAGUCCGUUAUCAACUUGAAAAAGUGGCACCGAGUCGGUGCUUUU
WDR20A guide #1	UCAAGAGUCAGUUCAGCACGGUUUUAGAGCUAGAAAUAGCAAGUUAAAAUAAGGCUAGUCCGUUAUCAACUUGAAAAAGUGGCACCGAGUCGGUGCUUUU
WDR20A guide #2	AACGUAAAUGUCAAAGAUGGGUUUUAGAGCUAGAAAUAGCAAGUUAAAAUAAGGCUAGUCCGUUAUCAACUUGAAAAAGUGGCACCGAGUCGGUGCUUUU
WDR20B guide #1	ACUCAAUUCAGCACCCGCGAGUUUUAGAGCUAGAAAUAGCAAGUUAAAAUAAGGCUAGUCCGUUAUCAACUUGAAAAAGUGGCACCGAGUCGGUGCUUUU
WDR20B guide #2	AUGAAUACUAAUAUCCAAGAGUUUUAGAGCUAGAAAUAGCAAGUUAAAAUAAGGCUAGUCCGUUAUCAACUUGAAAAAGUGGCACCGAGUCGGUGCUUUU

Supplementary Table I. Sequences (5’->3’) of sgRNAs used for zebrafish CRISPRi of USP46 complex components.
