# Supplementary material for: The USP46 complex deubiquitylates LRP6 to promote Wnt/β-catenin signaling
Source: Nat Commun. 2023 Oct 5;14:6173. doi: 10.1038/s41467-023-41836-z (PMC10556042; doi:10.1038/s41467-023-41836-z)
Supplement: Supplementary file 3 — Reporting Summary [file 41467_2023_41836_MOESM3_ESM.pdf]

nature portfolio

Corresponding author(s): Ethan Lee

Last updated by author(s): September 7, 2023

Reporting Summary

Nature Portfolio wishes to improve the reproducibility of the work that we publish. This form provides structure for consistency and transparency in reporting. For further information on Nature Portfolio policies, see our Editorial Policies and the Editorial Policy Checklist.

Statistics

For all statistical analyses, confirm that the following items are present in the figure legend, table legend, main text, or Methods section.

n/a	Confirmed	
☐	☒	The exact sample size (*n*) for each experimental group/condition, given as a discrete number and unit of measurement
☐	☒	A statement on whether measurements were taken from distinct samples or whether the same sample was measured repeatedly
☐	☒	The statistical test(s) used AND whether they are one- or two-sided<br>*Only common tests should be described solely by name; describe more complex techniques in the Methods section.*
☒	☐	A description of all covariates tested
☒	☐	A description of any assumptions or corrections, such as tests of normality and adjustment for multiple comparisons
☐	☒	A full description of the statistical parameters including central tendency (e.g. means) or other basic estimates (e.g. regression coefficient) AND variation (e.g. standard deviation) or associated estimates of uncertainty (e.g. confidence intervals)
☐	☒	For null hypothesis testing, the test statistic (e.g. *F*, *t*, *r*) with confidence intervals, effect sizes, degrees of freedom and *P* value noted<br>*Give P values as exact values whenever suitable.*
☒	☐	For Bayesian analysis, information on the choice of priors and Markov chain Monte Carlo settings
☒	☐	For hierarchical and complex designs, identification of the appropriate level for tests and full reporting of outcomes
☒	☐	Estimates of effect sizes (e.g. Cohen's *d*, Pearson's *r*), indicating how they were calculated

Our web collection on statistics for biologists contains articles on many of the points above.

Software and code

Policy information about availability of computer code

Data collection	TopFlash luciferase activity was collected by a FLUOstar Luminometer. Immunoblots were collected by a LI-COR Image Studio. Quantitative PCR data of HEK293T cells were collected by Bio-Rad CFX manager 3.1. Images of organoids were obtained using an Olympus IXSl inverted fluorescence microscope and Olympus CellSens software. Images of zebrafish embryos were obtained using a Zeiss Stemi 2000-CS microscope with an Olympus DP72 camera.
Data analysis	Data were analyzed with PRISM 9. Two-tailed student's t-test and Fisher's exact test, and one-way ANOVA followed by Tukey's multiple comparisons testwere done using Prism GraphPad 9. Statistical analyses were performed in R v3.1.0. Quantification of immunoblots was performed using ImageJ.

For manuscripts utilizing custom algorithms or software that are central to the research but not yet described in published literature, software must be made available to editors and reviewers. We strongly encourage code deposition in a community repository (e.g. GitHub). See the Nature Portfolio guidelines for submitting code & software for further information.

Data

Policy information about availability of data

All manuscripts must include a data availability statement. This statement should provide the following information, where applicable:

- Accession codes, unique identifiers, or web links for publicly available datasets
- A description of any restrictions on data availability
- For clinical datasets or third party data, please ensure that the statement adheres to our policy

All data supporting the findings of this study are available in the paper and the Supplementary information file. Raw data and original gel images are included in the Source Data file. All other relevant data are available from the authors upon reasonable request.

Research involving human participants, their data, or biological material

Policy information about studies with human participants or human data. See also policy information about sex, gender (identity/presentation), and sexual orientation and race, ethnicity and racism.

Reporting on sex and gender	N/A
Reporting on race, ethnicity, or other socially relevant groupings	N/A
Population characteristics	N/A
Recruitment	Formalin-fixed paraffin embedded normal tissue from de-identified subjects was obtained without clinical history or a link back to the original study subject. Unstained slides were collected and used for multiplex immunofluorescence.
Ethics oversight	De-identified normal human intestinal tissue was obtained through an IRB-approved Exempt Study (IRB #231475). Formalin-fixed paraffin embedded tissue from de-identified subjects was obtained without clinical history or a link back to the original study subject. Unstained slides were collected and used for multiplex immunofluorescence.

Note that full information on the approval of the study protocol must also be provided in the manuscript.

Field-specific reporting

Please select the one below that is the best fit for your research. If you are not sure, read the appropriate sections before making your selection.

☒ Life sciences ☐ Behavioural & social sciences ☐ Ecological, evolutionary & environmental sciences

For a reference copy of the document with all sections, see nature.com/documents/nr-reporting-summary-flat.pdf

Life sciences study design

All studies must disclose on these points even when the disclosure is negative.

Sample size	Sample size was predetermined based on prior use of experimental reagents and procedures. Ex vivo experiments were performed at least in triplicate and repeated three times. Nine measures per condition yield 84% power to detect a standardized difference (delta group averages/ common SD) of 1.5 based on a two-sample t-test with two-sided a=0.05. For zebrafish studies, at least 30 samples were included in each group. Sample size was determined consistently with previous publications in the field. These sample sizes ensure adequate statistical power as well as reproducibility of our assays.
Data exclusions	No data was excluded.
Replication	All experiments were performed at a minimum three times.
Randomization	Experiments were controlled by performing each one and subsequent reproducibility studies under constant conditions. Xenopus and zebrafish used in this study were randomly separated into each groups. For experiment using cell-lines or organoids, no specific passage of cells was required and cells were randomly separated into groups.
Blinding	Researchers were not blinded to ensure data are correctly collected from each treatment group. Experiments were also performed by other members in the lab and collaborators and replicated in their hands. Finally, the data were analyzed in a quantifiable fashion and statistical significance determined.

Reporting for specific materials, systems and methods

We require information from authors about some types of materials, experimental systems and methods used in many studies. Here, indicate whether each material, system or method listed is relevant to your study. If you are not sure if a list item applies to your research, read the appropriate section before selecting a response.

Materials & experimental systems

n/a	Involved in the study
☐	☒ Antibodies
☐	☒ Eukaryotic cell lines
☒	☐ Palaeontology and archaeology
☐	☒ Animals and other organisms
☒	☐ Clinical data
☒	☐ Dual use research of concern
☒	☐ Plants

Methods

n/a	Involved in the study
☒	☐ ChIP-seq
☒	☐ Flow cytometry
☒	☐ MRI-based neuroimaging

Antibodies

Antibodies used

Rat anti-HA (Roche, ROAHAHA)- (1:1000)
Mouse anti-human GAPDH (DSHB, hGAPDH-2G7) (1:500)
Mouse anti-beta-catenin (BD Transduction Laboratory, 610154) (1:1000 for immunoblots; 1:300 for immunofluorescence)
Mouse anti-Axin1 (CST, 2087) (1:1000)
Rabbit anti-LRP6 (CST, 2560) (1:1000)
Rabbit USP46 (Proteintech, 13502-1-AP) (1:1000 for immunoblots; 1:100 for immunofluorescence)
Rabbit anti-FLAG (Proteintech, 20543-1-AP) (1:2000)
Rabbit anti-WDR20 (Bethyl Laboratories, A301-657A) (1:2500 for immunoblots; 1:100 for immunofluorescence)
Rabbit anti-WDR48/ UAF1 (Proteintech, 16503-1-AP) (1:1000 for immunoblots; 1:50 for immunofluorescence)
Mouse anti-Tubulin (DSHB,E7) (1:1000)
Goat anti-mouse lgG H+L HRP (Promega, W4021) (1:5000)
Goat anti-Rabbit lgG H+L HRP (Promega, W4011)(1:5000)
Goat anti-rat lgG H+L HRP (Thermo, 31470) (1:5000)
Rabbit anti-Insulin Receptor beta (4B8) (CST, 3025) 1:1000
Goat anti-mouse Cy3 (Abcam ab97035) (1:100)
Goat anti-rabbit alexa fluor 647 (Invitrogen A-21245) (1:150)

Validation

We used all antibodies under manufacturers' recommended conditions and/or based on multiple publications:
Rat anti-HA https://www.sigmaaldrich.com/US/en/product/roche/roahaha;
Mouse anti-human GAPDH - https://dshb.biology.uiowa.edu/DSHB-hGAPDH-2G7;
Mouse anti-beta-catenin - https://www.bdbiosciences.com/en-us/products/reagents/microscopy-imaging-reagents/immunofluorescence-reagents/purified-mouse-anti-catenin.610154;
Mouse Anti-Axin1 - https://www.cellsignal.com/products/primary-antibodies/axin1-c76h11-rabbit-mab/2087;
Rabbit anti-LRP6 - https://www.cellsignal.com/products/primary-antibodies/lrp6-c5c7-rabbit-mab/2560;
Rabbit USP46 - https://www.ptglab.com/products/USP46-Antibody-13502-1-AP.htm;
Rabbit anti-FLAG - https://www.ptglab.com/products/Flag-Tag-Antibody-20543-1-AP.htm;
Rabbit anti-WDR20 - https://www.fortislife.com/products/primary-antibodies/rabbit-anti-wdr20-antibody/BETHYL-A301-657;
Rabbit anti-WDR48/ UAF1 - https://www.ptglab.com/products/WDR48-Antibody-16503-1-AP.htm;
Mouse anti-Tubulin (DSHB,E7) -https://dshb.biology.uiowa.edu/E7_2;
Goat Anti-mouse lgG H+L HRP - https://www.promega.com/products/protein-detection/primary-and-secondary-antibodies/anti_mouse-igg-h-and-l-hrp-conjugate/?catNum=W4021;
Goat Anti-Rabbit lgG H+L HRP - https://www.promega.com/products/protein-detection/primary-and-secondary-antibodies/anti-rabbit-igg-h-and-l-hrp-conjugate/?catNum=W4011;
Goat Anti-rat lgG H+L HRP - https://www.thermofisher.com/antibody/product/Goat-anti-Rat-IgG-H-L-Secondary-Antibody-Polyclonal/31470;
Rabbit anti-Insulin Receptor beta (4B8) -https://www.cellsignal.com/products/primary-antibodies/insulin-receptor-b-4b8-rabbit-mab/3025;
Goat anti-mouse Cy3 - https://www.abcam.com/products/secondary-antibodies/goat-mouse-igg-hl-cy3--preadsorbed-ab97035.html;
Goat anti-rabbit Alexa Fluor 647 - https://www.thermofisher.com/antibody/product/Goat-anti-Rabbit-IgG-H-L-Highly-Cross-Adsorbed-Secondary-Antibody-Polyclonal/A-21245

Eukaryotic cell lines

Policy information about cell lines and Sex and Gender in Research

Cell line source(s)

HEK293 (ATCC, CRL-1573)
HEK293STF (ATCC, CRL-3249)
DLD-1 (ATCC, CCL-221)
A172 and U87 (William Weiss Lab, UCSF)

Authentication

For ATCC lines, authentication was performed by the manufacturer.
HEK293 - https://www.atcc.org/products/crl-1573
HEK293STF - https://www.atcc.org/products/crl-3249

	DLD1 - https://www.atcc.org/products/ccl-221?nt=wobj-20-q<br>No authentication was performed for A172 and U87
Mycoplasma contamination	All cell lines were tested negative for mycoplasma contamination
Commonly misidentified lines<br>(See ICLAC register)	N/A

Animals and other research organisms

Policy information about studies involving animals; ARRIVE guidelines recommended for reporting animal research, and Sex and Gender in Research

Laboratory animals	Male and female Xenopus laevis (12-18 months); qRT-PCR studies used animal cap explants from stage 10.5 embryos (11 hours post fertilization at 23°C); Phenotyping used stage 35 embryos (2 day post fertilization at 23°C)<br><br>Male and female zebrafish (strain NHGRI) (~12 months)- qRT-PCR studies used 24 hours post fertilization (24 hpf at 28.5°C); Phenotyping used 3 days post fertilization (3 dpf at 28.5°C)
Wild animals	Not applicable
Reporting on sex	Sex was not considered a variable in the study.
Field-collected samples	No field-collected samples were used in this study.
Ethics oversight	All animals in this study (Xenopus and zebrafish) were treated in accordance with (and approval from) Vanderbilt University's and the University of Maryland School of Medicine's Institutional Animal Care and Use Committees.

Note that full information on the approval of the study protocol must also be provided in the manuscript.
